# Draft genome sequence of *Bacillus thuringiensis* serovar. toumanoffi NRRL B-4059: identification of a novel Cry protein candidate

**DOI:** 10.1128/mra.00143-24

**Published:** 2024-11-14

**Authors:** Robert W. Murdoch, Bryan T. Gemler, Nathan Wallace, Bradley S. Heater

**Affiliations:** 1 Battelle Memorial Institute, Columbus, Ohio, USA; Indiana University, Bloomington, Bloomington, Indiana, USA

**Keywords:** *Bacillus thuringiensis*, Cry protein

## Abstract

Here, we report the high-quality draft genome of *Bacillus thuringiensis* serovar. toumanoffi NRRL B-4059, with a complete circular chromosome and nine circular plasmids. The assembly is 7,031,170 bp and encodes 6,974 protein-coding genes, 42 rRNA genes, 107 tRNA genes, and has 34.7% GC content. Nine genes encoding Cry proteins were identified, including one previously unidentified Cry protein candidate.

## ANNOUNCEMENT

*Bacillus thuringiensis* naturally produces insecticidal Cry protein toxins and has been instrumental in the development of pesticides (1). Recently, insect pests have gained resistance against traditional Cry proteins (2), so the elucidation of novel Cry toxin candidates is warranted. Strains of *Bacillus thuringiensis* serovar. toumanoffi have been shown to generate more than five Cry proteins (3), but the genomic characterization of this serotype group has thus far been limited.

B-4059 was obtained from the United States Department of Agriculture (USDA) Northern Regional Laboratory (NRRL) Culture Collection (accession number B-4059). NRRL reports that B-4059 was originally isolated from a diseased insect and has been indicated as a serotype toumanoffi (4). B-4059 was streaked onto tryptic soy agar and incubated for 18 h at 30°C, followed by growth in brain heart infusion broth for 18 h at 30°C and 200 RPM. DNA was extracted and sequenced by the Genomics and Microbiome Core Facility at Rush University. Cells were lysed using a Claremont Bio OMNILYSE and DNA was extracted per the Promega Maxwell RSC Cultured Cells DNA Kit protocol, sheared by multiple passes through a pipette tip, and size selected by 0.75% agarose gel purification. PacBio HiFi library was prepared from 2 µg of sheared and size-selected DNA (10 kb to 20 kb) with SMRTBell Express Template Prep kit 2.0. Sequencing was performed on an SMRT cell on a Pacbio Sequel Ile using 30 hs movie time. Sequencing generated 2,490,932 reads with an N50 of 6.3 Kbp and 195,133 reads over 10 Kbp in length. Reads were quality trimmed and assembled using Canu version 2.2 (5, 6) operated with default settings and circularity detection followed by removal of bubble and repeat contigs. Contigs flagged as circular were not subjected to end-trimming or rotation.

The assembly (34.7% GC) consisted of 11 contigs, the largest of which was 5.63 Mbp and circular. Nine circular plasmids ranging from 23.6 Kbp to 556 Kbp were also identified.

The assembly was annotated using Bakta v1.5.1 (7). Taxonomic placement of the 14 16S rRNA genes, achieved by sequence query against the SILVA Ref NR database using the SINA align and classify web server operated under default parameters (8, 9), was consistent with the *Bacillus* or *B. cereus* group.

Cry candidates were detected (10) and then classified using the Bacterial Pesticidal Protein Resource Center (BPPRC) (11). Nine Cry proteins were identified, one of which (BTTOUR_02245) represents a novel member of the Cry1 group (Table 1). Given the low sequence homology to known Cry proteins, BTTOUR_02245 could be a potential candidate to screen for insecticidal activity against Cry-resistant pests.

**TABLE 1:** Putative Cry proteins detected in the *Bacillus thuringiensis* serovar. toumanoffi NRRL B-4059 genome, as determined by their Pfam domains and The Bacterial Pesticidal Protein Resource Center database and classification system

Locus tag	Pfam domains	BPPRC top match result(s) (percentage Identity)	BPPRC classification	BPPRC top percent identity
BTTOUR_33045	Endotoxin_N, Endotoxin_M	Cry1Ab18 (99.9) Cry1Ab24 (99.9) Cry1Ab27 (97.6)	Cry1Ab	99.9
BTTOUR_33105	Endotoxin_N, Endotoxin_M	Cry1Ja2 (99.9) Cry1Ja3 (99.8) Cry1Ja1 (99.5)	Cry1Ja	99.9
BTTOUR_33130	Endotoxin_N, Endotoxin_M	Cry1Id1 (99.9) Cry1Id2 (99.9) Cry1Id3 (99.3)	Cry1Id	99.9
BTTOUR_33135	Endotoxin_N, Endotoxin_M	Cry1Da3 (99.8) Cry1Da5 (99.7) Cry1Da4 (99.5)	Cry1Da	99.8
BTTOUR_33315	Endotoxin_N	Cry2Ad1 (99.8) Cry2Ad6 (99.4) Cry2Ad2 (99.2)	Cry2Ad	99.8
BTTOUR_33080	Endotoxin_N, Endotoxin_M	Cry1Nb1 (99.1) Cry1Na3 (76.5) Cry1Na2 (76.4)	Cry1Nb	99.1
BTTOUR_33010	Endotoxin_N, Endotoxin_M	Cry1Bb1 (98.6) Cry1Bb2 (96.6) Cry1Bb3 (96.5)	Cry1Bb	98.6
BTTOUR_33000	Endotoxin_N, Endotoxin_M	Cry1Hb1 (98.2) Cry1Hb2 (98.0) Cry1Ha1 (86.9)	Cry1Hb	98.2
BTTOUR_02245	Endotoxin_N, Endotoxin_M	Cry1Ma2 (38.7) Cry3Aa12 (38.1) Cry3Aa13 (38.1)	Cry1	38.7

## Data Availability

The complete genome sequence of *Bacillus thuringiensis* serovar. toumanoffi NRRL B-4059 has been deposited at DDBJ/EMBL/GenBank under accession numbers PRJNA1034086 (BioProject), SAMN37927671 (BioSample), SRR26621005 (SRA), and GCA_033663765 (GenBank).
